# SERIES: eHealth in primary care. Part 4: Addressing the challenges of implementation

**DOI:** 10.1080/13814788.2020.1826431

**Published:** 2020-10-07

**Authors:** Anke Versluis, Sanne van Luenen, Eline Meijer, Persijn J. Honkoop, Hilary Pinnock, David C. Mohr, Ana Luisa Neves, Niels H. Chavannes, Rianne M. J. J. van der Kleij

**Affiliations:** a Department of Public Health and Primary Care, Leiden University Medical Centre, Leiden, the Netherlands; b National eHealth Living Lab (NeLL), Leiden, the Netherlands; c Section of Clinical Psychology, Institute of Psychology, Faculty of Social and Behavioural Sciences, Leiden University, Leiden, the Netherlands; d Usher Institute, University of Edinburgh, Edinburgh, UK; e Center for Behavioral Intervention Technologies, Department of Preventive Medicine, Northwestern University, Chicago, IL, USA; f Institute of Global Health Innovation, Imperial College London, London, UK; g Center for Health Technology and Services Research (CINTESIS)/Department of Community Medicine, Health Information and Decision (MEDCIDS), Faculty of Medicine, University of Porto, Porto, Portugal

**Keywords:** eHealth, primary care, implementation, barriers, facilitators

## Abstract

**Background:**

The implementation of eHealth applications in primary care remains challenging. Enhancing knowledge and awareness of implementation determinants is critical to build evidence-based implementation strategies and optimise uptake and sustainability.

**Objectives:**

We consider how evidence-based implementation strategies can be built to support eHealth implementation.

**Discussion:**

What implementation strategies to consider depends on (potential) barriers and facilitators to eHealth implementation in a given situation. Therefore, we first discuss key barriers and facilitators following the five domains of the Consolidated Framework for Implementation Research (CFIR). Cost is identified as a critical barrier to eHealth implementation. Privacy, security problems, and a lack of recognised standards for eHealth applications also hinder implementation. Engagement of key stakeholders in the implementation process, planning the implementation of the intervention, and the availability of training and support are important facilitators. To support care professionals and researchers, we provide a stepwise approach to develop and apply evidence-based implementation strategies for eHealth in primary care. It includes the following steps: (1) specify the eHealth application, (2) define problem, (3) specify desired implementation behaviour, and (4) choose and (5) evaluate the implementation strategy. To improve the fit of the implementation strategy with the setting, the stepwise approach considers the phase of the implementation process and the specific context.

**Conclusion:**

Applying an approach, as provided here, may help to improve the implementation of eHealth applications in primary care.

## eHealth in primary care

KEY MESSAGES

- To successfully implement eHealth in primary care, context-specific implementation strategies are essential.
- Identifying potential barriers (e.g. costs) and facilitators (e.g. support) to eHealth implementation is necessary to develop the right implementation strategy.
- The provided tool helps to define the implementation problem and desired implementation behaviour and develop evidence-based implementation strategies.

As the population ages and multimorbidity becomes the norm [1], the workload of healthcare professionals in primary care not only increases but also becomes more complex. As a result, healthcare needs to be organised more efficiently to ensure that both the quality of care and the wellbeing of professionals are maintained [1]. eHealth, defined as ‘health services and information delivered or enhanced through the Internet and related technologies’ [2], might be one of the solutions to improve the efficiency of care [3]. The potential benefits of the uptake of eHealth in primary care are diverse and can include a reduction of errors and costs, increased productivity of primary care physicians [4], and improved disease management of patients [5]. However, eHealth may also have some disadvantages, for example, it may feel less personal than face-to-face care and health inequalities may be increased because users often need a certain degree of (health) literacy and digital skills [6]. EHealth applications can be classified following the conceptual model of Shaw et al. [7]. It distinguishes: (1) eHealth to monitor health parameters such as the number of steps and sleep quality; (2) eHealth to communicate between stakeholders, e.g. patients and healthcare professionals, and (3) eHealth for the collection, analysis, and management of data, e.g. *via* electronic health records. The model and the potential of eHealth are described in more detail in the first article of this series [6].

This background paper discusses the implementation of eHealth in primary care. The primary purpose of this paper is to discuss strategies to optimise the implementation of eHealth and to provide a practical, step-by-step approach to develop evidence-based implementation strategies that fit the specific context. What implementation strategies to consider, however, depends on (potential) barriers and facilitators in a given situation. Therefore, after an introduction on the implementation of eHealth interventions in primary care, we give a brief overview of key barriers and facilitators to eHealth implementation. Knowing what potential barriers and facilitators can be at play, could help researchers, clinicians, and policymakers develop the right implementation strategy.

## Implementation of interventions in primary care

A translational gap between research and practice exists, meaning that proven effective healthcare innovations often do not reach practice or only after a significant length of time. Moreover, the implementation of new interventions into healthcare is not always successful [8]. This is alarming as it can affect the quality of care being delivered [8]; it may, for example, harm the effectiveness and safety of healthcare. This highlights that there is room for improvement. Implementation research is specifically focussed on methods to promote the uptake of research findings and other evidence-based practices into routine practice and thereby aims to improve the quality of healthcare [9].

**Figure 1.**
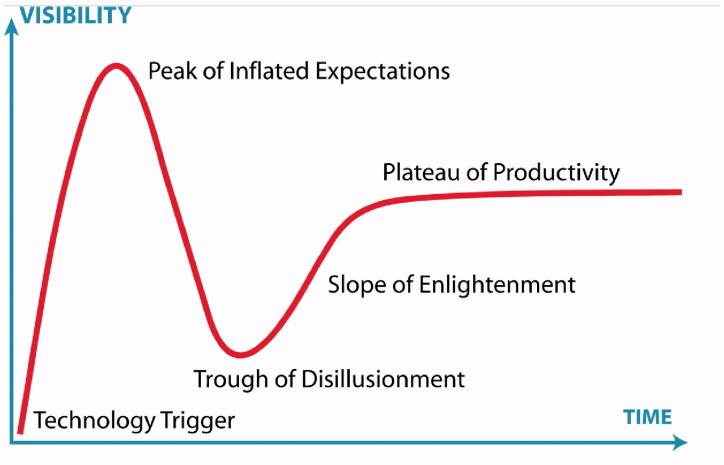
Hype cycle of Gartner. Source: https://en.wikipedia.org/wiki/Hype_cycle.

The hype cycle of Gartner can be used to determine at what stage of uptake an eHealth application is at any given point in time [10]. As shown in Figure 1, the visibility or use of technological innovation rapidly increases after the introduction because of inflated expectations about the innovation. Next, there is a decline in uptake as the real-world challenges with the implementation come to light (i.e. Trough of Disillusionment). When, at this stage, the shortcomings or obstacles to successful implementation are addressed, the uptake of the innovation can be boosted (i.e. Slope of Enlightenment) and reach a stable level. As mentioned in the first article of this series [6], it is essential to take the interaction between end-user demands, technology, and context into account to sustain the use of the eHealth innovation.

The success rate of eHealth implementation is unknown; however, many examples of eHealth implementation failures have been described [11]. Next to the translational gap between research and practice, there also seems to be an evidence gap. Many new eHealth applications are not yet investigated on effectiveness [6,12]. For example, a scoping review on eHealth applications for people with COPD showed that most eHealth applications were not thoroughly investigated on effectiveness [13]. The evidence of effectiveness is, however, a basic requirement for implementation [14,15]. The phase between adopting an eHealth application and the routine use of the application is multidimensional and complex [15]. It is therefore vital to gain knowledge on which factors impact eHealth implementation, and how we can effectively target these factors. Several different barriers and facilitators that can influence the implementation success have been identified. The framework for non-adoption, abandonment, scale-up, spread and sustainability (NASSS) of healthcare technologies posits that the implementation of eHealth applications will only succeed when the different interacting domains are acknowledged and addressed (e.g. characteristics of the technology, end-user, organisation and outer setting) [16]. When effective eHealth applications are successfully implemented in routine care, this may benefit the quality of healthcare.

## An implementation framework

**Figure 2.**
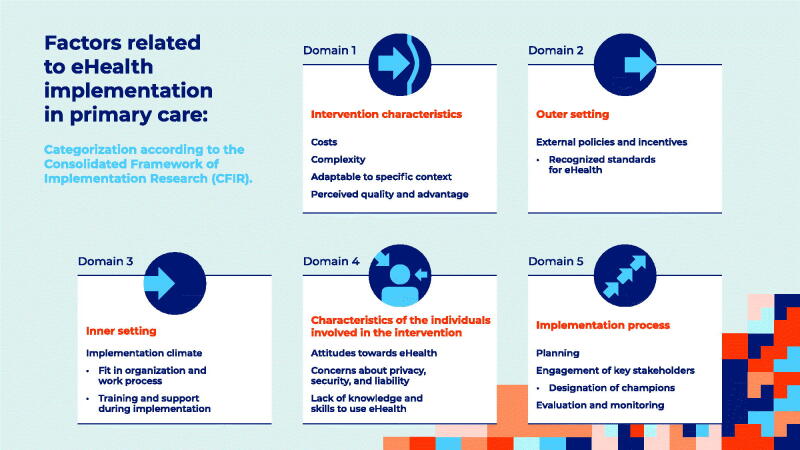
Overview of the different domains related to eHealth implementation in primary care, including several potential barriers and facilitators per domain.

The Consolidated Framework for Implementation Research (CFIR) is widely used to categorise barriers and facilitators to implementation [17]. The CFIR is a theory-driven model and comprises five domains: (1) the intervention characteristics, (2) the outer setting, (3) the inner setting, (4) the characteristics of the individuals involved in the intervention, and (5) the implementation process. In Supplementary Appendix 1, we describe each domain and list critical barriers and facilitators to eHealth implementation that were reported in the literature and give examples of how barriers and facilitators can affect the implementation of eHealth. The appendix does not provide an exhaustive list of all potential barriers and facilitators but offers insight into the most essential and changeable implementation factors. This inventory will help to determine what obstacles need to be overcome and how we might optimise eHealth implementation in primary care. It is important to realise that certain factors can be considered both a facilitator and a barrier. For example, financial costs are frequently mentioned as a factor affecting eHealth implementation [e.g. 15,18,19,20]. When there are high costs and there is no or limited funding, financial cost is a barrier; however, low costs and the availability of funding can be considered a facilitator. Figure 2 presents an overview of the domains and the key factors influencing eHealth implementation.

## Implementation strategies

To enable the successful implementation of eHealth applications in practice, the correct implementation strategies must be chosen. Implementation strategies refer to the ‘methods or techniques used to enhance the adoption, implementation, and sustainability of a clinical programme or practice’ [21]. A comprehensive list of 73 implementation strategies is provided by Powell et al. [22] (e.g. promote adaptability, centralise technical assistance, identify and prepare champions). Strategies can be used individually or combined into a multi-component implementation approach, which allows users to target the different relevant domains simultaneously (e.g. organisational, individual, or policy level). When choosing implementation strategies, it is crucial to ensure that they fit the phase of the implementation process (i.e. adoption phase vs implementation phase) and the specific context (e.g. characteristics of the GP practice and stakeholders involved). More specific, certain implementation strategies may be better suited for the adoption phase of implementation (e.g. developing a formal implementation blueprint). In contrast, other strategies can better be applied when implementation has already started (e.g. identifying early adopters).

To facilitate healthcare professionals and researchers who are in the process of implementing an eHealth intervention, we have provided a practical worksheet to effectively target expected or experienced barriers and facilitators (Supplementary Appendix 2; Table 1). The worksheet is based on the Action, Actor, Context, Target, Time (i.e. AACTT) framework, which helps to specify what behaviour needs to change and how to define this particular behaviour [23]. The worksheet contains the following steps: (1) specify the eHealth application, (2) define the problem, (3) specify the desired implementation behaviour, i.e. describe ‘who does what; to, for or with whom; when; where?’ [23], (4) choose the implementation strategy from the overview provided by Powell et al. [22], and (5) evaluate the implementation strategy. A description of each step is given in Table 1 and examples are given in Supplementary Appendix 2. The barriers and facilitators to eHealth implementation that are described in Supplementary Appendix 1 may be used to fill in the worksheet. More specifically, the determinants may be used to help define the problem in step 2 of the worksheet and they may guide the choice of the implementation strategy in step 4. For example, a problem may be that professionals were not adequately trained to work with a new eHealth application (see domain 3: Inner setting). Conducting ongoing training may be an implementation strategy to help overcome this problem. The worksheet can be a practical tool to facilitate the development and application of evidence-based implementation strategies. The worksheet takes into account the phase of the implementation process and the specific context in which the eHealth application is implemented, and this may improve the fit of the implementation strategy with the setting. Besides developing evidence-based implementation strategies, it is important to be aware of the ethical implications of the implementation of eHealth in primary care (e.g. questions related to roles and responsibilities). This topic is further discussed in the second article of this series [24].

**Table 1.** A stepwise tool to build an evidence-based implementation strategy.

Step	Explanation
1. Specify eHealth application	Which eHealth application do you want to implement? What is the goal of the eHealth application?
2. Define problem	What implementation problems do you anticipate or have you encountered? And which of these problems is most important and changeable?
3. Specify desired implementation behaviour	
3a. Action	A concrete, observable behaviour to address the implementation problem.
3b. Actor	The individual or group that will act.
3c. Context	The physical, emotional or social setting in which the actor will act.
3d. Target	The individual or group for/with/on behalf of whom the actor will act.
3e. Time	The time and duration that the actor will perform the action with/for the target.
4. Choose implementation strategy	Choose the implementation strategy of the list of Powell et al. [22] that fits best. Choosing an implementation strategy that fits with the phase of the implementation process (i.e. adoption phase vs implementation phase).
5. Evaluate implementation strategy	Evaluate the implementation of the eHealth application.

*Note.* Based on the action, actor, context, target, time (AACTT) framework of Presseau et al. [23].

## Conclusion

The implementation of eHealth applications in primary care is challenging. Broadening knowledge on barriers and facilitators that influence the implementation of eHealth applications is essential to promote successful uptake and maintenance. Cost, privacy, security problems, and a lack of recognised standards for eHealth applications were identified as important barriers, whereas engaging stakeholders, planning the implementation, and the availability of training and support were considered facilitators. To allow eHealth applications to be successfully implemented, it is important that context-specific implementation strategies are applied that are in line with the phase of the implementation process. The step-by-step worksheet provided may help researchers and healthcare professionals who are implementing an eHealth application, to develop and apply evidence-based implementation strategies.

## Supplementary Material

Supplemental Material - Appendix 1

Supplemental Material - Appendix 2
