# Supplementary material for: SERIES: eHealth in primary care. Part 4: Addressing the challenges of implementation
Source: Eur J Gen Pract. 2020 Oct 7;26(1):140–5. doi: 10.1080/13814788.2020.1826431 (PMC7580793; doi:10.1080/13814788.2020.1826431)
Supplement: Supplemental Material - Appendix 1 [file IGEN_A_1826431_SM9831.pdf]

Appendix 1. Description of the domains of the Consolidated Framework for Implementation Research (CFIR), and critical barriers and facilitators to eHealth implementation.

CFIR domain plus associated barriers and facilitators	Description
**Domain 1. Intervention characteristics**	**Characteristics of the eHealth application that influence implementation**
Costs [15,18-20]	The absence of financial support can be a barrier to implementation. Costs can be related to the start-up of eHealth applications (e.g., purchasing), to ongoing costs (e.g., IT support), or both. The start-up costs can be high because eHealth applications are often developed by start-ups that have made considerable financial investments. These start-ups may, therefore, not always want to share the content of their application in case of success. Funding, financial incentives or new business models may be useful to overcome this barrier.
Complexity [14,15,18]	Complex eHealth applications, like software that is not intuitive and therefore not user-friendly, can be a barrier. To circumvent complexity issues, eHealth technology should—first and foremost—be reliable, fast and easy to use.
Adaptable to specific context [15]	eHealth applications need to be adaptable to the specific context of the HCP, i.e., static applications can be a barrier to implementation.
Perceived quality and advantage [4,19]	Negative expectations about the quality of the eHealth application (e.g., whether it is acceptable and will have the desired outcome) and about the (added) advantage can be considered a barrier to implementation. Positive expectations about quality and advantage can be considered a facilitator.

Example of *cost* as a barrier to implementation	In 2008 NHS Lothian implemented a telemonitoring service for people with COPD in the UK. The service enabled patients to record their symptoms online, perform physiological measurements, and transmit their health data to trained support staff [25]. Minor technical issues (such as batteries needing replacing in peripherals) caused problems because the service was set-up without the availability of technical support. The clinical team thus had to spend valuable time resolving technical issues. Although ultimately resolved, the cost of ongoing technical support resulted in unanticipated costs that were not budgeted. It is essential to budget adequately for ongoing support costs, mainly when eHealth is provided for people unfamiliar with technology.
Example of *complexity* as a barrier to implementation	eVita is a personal health record, including self-management support and coaching for patients with diabetes type II in primary care [26]. The eHealth tool had low usage and only 27% of those registered to use the service logged in at least once. It was found that the complexity of the login procedure was a barrier for patients to use the eHealth application. A perceived facilitator, for HCP, was the ease with which the helpdesk could be contacted.
Example of a successful implementation facilitated by intervention characteristics	The mass uptake of a remote consultation service, in the UK, in response to the COVID-19 pandemic was made possible by the availability of a simple, easy-to-use service [27]. See also the second example under domain 2. The positive experiences of HCP with this service may, in turn, positively affect the perceived advantage of using eHealth and promote the uptake of other eHealth services in the future.

**Domain 2. Outer setting**	**Aspects external to the organization**
External policies and incentives [14,15,18-20]	There are, for example, concerns about the return on investment of eHealth applications (i.e., achieved benefits relative to investment costs). These concerns may be explained by the fact that costs related to eHealth implementation are generally not reimbursed, but often need to be covered by the healthcare organisation.
Lack of recognised standards for eHealth [15,18]	Recognised standards for eHealth applications are missing (i.e., technology standards addressing the operability between systems, security and privacy) and this can be considered a barrier. The availability of such standards can impact concerns of HCP related to data safety and professional liability.
Examples of a successful implementation facilitated by external policies and incentives	Primary care practices in the UK could join a reimbursement program that incentivised certain aspects of quality care [28]. Indicators were, for example, in clinical areas (e.g. heart failure, asthma), in organisational areas (e.g. education), or related to patient experience of care. Crucially, the practice achievements were assessed via the EHR. Almost all practices voluntarily joined the initiative and most converted to paperless practice (from their previous hybrid status) over a few months. Although not the primary aim of the program, such a significant policy initiative was able to provide the context for the universal implementation of the EHR in UK primary care.<br><br>In response to the COVID-19 pandemic, a significant shift has occurred in primary care from face-to-face consultations to remote consultations (especially video consultations). This was important to

	minimise the risk of infection for patients and healthcare professionals. A new remote consultation service was quickly developed in the UK and 80 percent of the practices were using it [27]. Although the technology was ignored/resisted for years, it was widely accepted and adopted within weeks when it was necessary. The same happened in many other countries within and outside Europe.
**Domain 3. Inner setting**	**Characteristics of the organisation that implements the eHealth application**
Fit in organisation and work processes [15,17,20,29]	To be successfully implemented, the introduced eHealth application must be integrated into the organisation and its work processes. When the implemented application, for instance, disrupts the care delivery or perceived care delivery, it will negatively impact implementation success and risk increasing the workload of the HCP.
Training and support [15,19]	Implementation of eHealth can be facilitated when the management of an organisation supports it, and when there is the capacity to implement the innovation. There should be (a) enough time for HCP to (learn to) work with the new application, (b) available high quality and easily accessible training, and (c) support during implementation for HCP and patients (both technical and educational).
<u>Example of a successful implementation facilitated by the involvement of the organisation's management</u>	The MasterMind project aimed to make high-quality treatment for depression widely available using eHealth. Computerised Cognitive Behavioural Therapy and video conference for collaborative care were implemented in primary and specialised care across 10 EU and associated countries. The project was a success with 11,573 patients receiving eHealth treatment for depression, and the

	satisfaction among patients and HCP was high. In addition, the majority of patients showed an increase in quality of life and a reduction in depressive symptoms. The project showed that it is vital to involve the management of the organisation in the implementation of eHealth. In the project, this involvement provided resources for training and knowledge sessions for professionals and time to receive support [30].
**Domain 4. Characteristics of the individuals involved in the intervention**	**Characteristics of individuals who implement the eHealth application and the individuals who are targeted by the application**
Attitudes and beliefs [15,20,31]	Positive attitudes, like assumed benefit of eHealth for patients, is a facilitator. Negative attitudes, like the belief that eHealth will negatively affect the relation with the patient, can be a barrier.
Concerns about privacy, security, and liability [15,19]	Can act as a barrier and these concerns can be present in both patients and HCP.
Lack of knowledge and skills [15,19]	Can act as a barrier amongst patients and HCP, and limit acceptance and implementation.
**Domain 5. Implementation process**	**Refers to the process of implementation and includes planning, executing, reflecting, and evaluating**
Planning [15,31]	An incremental approach, where implementation in complex organisations takes place according to a plan, has been found to be preferable to a 'big bang' approach, where eHealth is implemented

	quickly and immediately. There are exceptions to this general rule, see for example the examples under domain 2.
Engagement of key stakeholders [14,15,31]	Engaging stakeholders in the development and implementation of eHealth can be considered a facilitator. This engagement may lead to a feeling of ownership of, confidence in, and acceptance of the eHealth application. The designation of champions can facilitate implementation. Champions may promote the eHealth application to more sceptical colleagues.
Evaluation and monitoring [14,15]	Evaluating and monitoring are important to ensure the benefits of the eHealth application, which may, in turn, lead to increased acceptance among stakeholders and ongoing funding. A lack of well-designed evaluation studies on eHealth applications may be considered as insufficient evidence and jeopardise support for the implementation of eHealth.
Example of lack of engagement of stakeholders as a barrier to implementation	On April 5, 2011, the Dutch government unanimously rejected the proposed legal regulation of a centralised, everywhere accessible EHR [32]. The government believed that there was too much confusion around the EHR to accept the Act of Parliament. In hindsight, the strategy that was used to implement the EHR was regarded as problematic. The Ministry of Health, Welfare and Sport took the lead in the realisation of the EHR and attempted to involve various stakeholders in the development process, such as the national GP association and the data protection college. In practice, however, this turned out differently than intended and, apart from the ministry, there was hardly anyone committed in the healthcare field. The ministry failed to bring the stakeholders together; the opinions were too different which resulted in a rejection of the bill regarding the EHR.

Note. COPD = chronic obstructive pulmonary disease; EHR = electronic health record; HCP = healthcare professionals.

References (numbers [1]-[24] can be found in the main article)

[25] Ure J, Pinnock H, Hanley J, et al. Piloting tele-monitoring in COPD: a mixed methods exploration of issues in design and implementation. Prim Care Respir J. 2012;21:57-64.

[26] Sieverink F, Braakman-Jansen A, Roelofsen Y, et al. The Diffusion of a Personal Health Record for Patients with Type 2 Diabetes Mellitus in Primary Care. Int J Adv Life Sci. 2014;6:177-183.

[27] Thornton J. Covid-19: how coronavirus will change the face of general practice forever. Br Med J. 2020;368.

[28] Doran T, Fullwood C, Gravelle H, et al. Pay-for-performance programs in family practices in the United Kingdom. N Engl J Med. 2006;355:375-384.

[29] Greenhalgh T. Role of routines in collaborative work in healthcare organisations. Br Med J. 2008;337:a2448.

[30] Pedersen CD, Craggs MA, Skjoth MM, et al. MasterMind Final Evaluation Report 2017. Place: MasterMind; 2017. [cited 2020 Sept 15]. Available from: http://mastermind-project.eu/wp-content/uploads/2018/02/MasterMind-D3.5-v1.0_-Final-evaluation-report.pdf

[31] Ludwick DA, Doucette JJIjomi. Adopting electronic medical records in primary care: lessons learned from health information systems implementation experience in seven countries. Int J Med Inform. 2009;78:22-31.

[32] van Twist MJW, Schulz JM, Chin-A-Fat NMH, et al. Het EPD voorbij? Evaluatie Besluitvormingsproces Kaderwet Elektronische Zorginformatie-uitwisseling. Place: Nederlandse School voor Openbaar Bestuur; 2012. [cited 2020 Sept 15]. Available from: https://www.parlementairemonitor.nl/9353000/1/j9vvij5epmj1ey0/viwtipyur4pu
