# Supplementary material for: SERIES: eHealth in primary care. Part 4: Addressing the challenges of implementation
Source: Eur J Gen Pract. 2020 Oct 7;26(1):140–5. doi: 10.1080/13814788.2020.1826431 (PMC7580793; doi:10.1080/13814788.2020.1826431)
Supplement: Supplemental Material - Appendix 2 [file IGEN_A_1826431_SM9411.pdf]

Appendix 2.

Worksheet. A stepwise tool to build an evidence-based implementation strategy.

<table>
<tr><th colspan="2">Step</th><th>Explanation</th><th>Answer</th></tr>
<tr><td colspan="2">Specify eHealth application</td><td>Which eHealth application do you want to implement?
What is the goal of the eHealth application?
E.g. That patients can make appointments digitally, increasing access of care.</td><td></td></tr>
<tr><td colspan="2" rowspan="2">Define problem</td><td>What implementation problems do you anticipate or have you encountered?
E.g. Financial problems and busy healthcare professionals.</td><td></td></tr>
<tr><td>Which of these problems is most important and changeable?
E.g. Financial problems.</td><td></td></tr>
<tr><td rowspan="2"></td><td>Action</td><td>A concrete, observable behaviour to address the implementation problem.
E.g. Search for funding within the government or forming collaborations with doctors from other practices.</td><td></td></tr>
<tr><td>Actor</td><td>The individual or group that will perform the action.
E.g. Researcher or healthcare professional.</td><td></td></tr>
</table>

Specify desired implementation behaviour	Context	The physical, emotional or social setting in which the actor will perform the action.<br>*E.g. Doctor's office or at a patient's home.*	
	Target	The individual or group for/with/on behalf of whom the actor will perform the action.<br>*E.g. Heart failure patients or GPs.*	
	Time	The time and duration that the actor will perform the action with/for the target.<br>*E.g. Once per week for 30 minutes during 3 months.*	
Choose implementation strategy		Choose the implementation strategy of the list of Powell et al. 2015 that fits best. Choosing an implementation strategy that fits with the phase of the implementation process (i.e., adoption phase vs implementation phase).<br>*E.g. Conduct ongoing training.*	
Evaluate implementation strategy		Evaluate the implementation of the eHealth application.<br>*E.g. Adaptation of the strategy is needed.*	

Note. Based on the Action, actor, context, target, time (AACTT) framework of Presseau et al. 2019
